# Prognostic differences among patients with idiopathic interstitial pneumonias with acute exacerbation of varying pathogenesis: a retrospective study

**DOI:** 10.1186/s12931-019-1247-z

**Published:** 2019-12-18

**Authors:** Motoyasu Kato, Tomoko Yamada, Shunichi Kataoka, Yuta Arai, Keita Miura, Yusuke Ochi, Hiroaki Ihara, Ryo Koyama, Shinichi Sasaki, Kazuhisa Takahashi

**Affiliations:** Department of Respiratory Medicine, Juntendo University Graduate School of Medicine, 3-1-3, Hongo, Bunkyo-ku, Tokyo, 113-8431 Japan

**Keywords:** Idiopathic interstitial pneumonias, Idiopathic pulmonary fibrosis, Acute exacerbation, Trigger, Infection

## Abstract

**Background:**

Acute exacerbation of chronic fibrosing idiopathic interstitial pneumonias (AE-IIPs) is associated with a high mortality rate. In 2016, an international working group proposed a revised diagnostic criteria for AE-IIPs, suggesting that it be classified as idiopathic or triggered. Many factors are known to trigger AE-IIPs, including surgery, infection, and drugs. However, it is unknown which AE-IIPs triggers have a worse prognosis. We aimed to investigate the prognosis of patients with various clinical types of AE-IIPs, particularly infection-triggered, non-infection triggered, and idiopathic AE-IIPs.

**Methods:**

We retrospectively collected data from 128 chronic fibrosing IIPs (CF-IIPs) patients who were hospitalized by respiratory failure between April 2009 and March 2019 at Juntendo University Hospital. Among these patients, we evaluated 79 patients who developed AE-IIPs and 21 who developed pneumonia superimposed on CF-IIPs. Patients with AE-IIPs were classified into three types: idiopathic, infection-triggered, and non-infection-triggered AE-IIPs. We analyzed differences in patient characteristics, examination findings; level of serum markers, results of pulmonary function, and radiological findings, prior treatment for baseline CF-IIPs, and prognosis. We then evaluated the risk factor for early death (death within 30 days from the onset of AE-IIPs) associated with AE-IIPs.

**Results:**

Among the patients who developed AE-IIPs, 34 were characterized as having idiopathic, 25 were characterized as having infection-triggered, and 20 were categorized as having non-infection-triggered AE-IIPs. Survival time for pneumonia superimposed on IIPs was significantly longer than that for any AE-IIPs. Survival time for bacterial pneumonia superimposed on CF-IIPs was significantly longer than that for AE-IIPs (for each idiopathic and all triggered IIPs). Thereafter, survival time for infection-triggered was significantly longer than for idiopathic or non-infection-triggered AE-IIPs. The mortality rate was significantly lower in infection-triggered AE-IIPs than in other types of AE-IIPs. Furthermore, the incidence of infection-triggered AE-IIPs in winter was significantly higher than that in other seasons. Moreover, the clinical AE-IIPs types and radiological findings at AE-IIP onset were significant risk factors for AE-IIPs-induced early death.

**Conclusions:**

Our findings suggest that patients with infection-triggered AE-IIPs can expect a better prognosis than can patients with other clinical types of AE-IIPs.

## Background

Acute exacerbation of idiopathic pulmonary fibrosis (AE-IPF) was first reported in 1993 [1]. The incidence of AE is reported to be approximately 10–20% in IPF [2]. Furthermore, AE-IPF is known to be associated with a high mortality rate of approximately 30–50% [3].

Diagnostic criteria for AE-IPF were first published in 2007 [4]. These included a previous diagnosis of interstitial pneumonia, including IPF, unexplained worsening of dyspnea in the past month, high resolution computed tomography (HRCT) evidence of new bilateral ground-glass opacities (GGO) or consolidation, and exclusion of alternative reasons for exacerbation, including pulmonary infection, and heart or renal failure. Recently, there have been descriptions of AE associated with other progressive fibrosing interstitial pneumonias, including non-specific interstitial pneumonia, chronic hypersensitivity pneumonia, and connective tissue disease-related interstitial pneumonia [5–9]. Patients with AE associated with these conditions have a worse prognosis, and it is believed to reflect disease progression in both progressive fibrosing interstitial pneumonias.

In 2016, Collard et al. published an International Working Group Report on AE-IPF [2]. This report suggested that clinical AE-IPF types should be categorized as “idiopathic” or “triggered.” Known triggers of AE include infection, surgery, medication, any biopsy, and bronchoscopy [10–17]. Despite this categorization, it is not known which of the two subtypes of AE have a worse prognosis. Moreover, we focus on differences between AE triggers, especially the difference between infective and non-infective triggers (e.g., drugs, surgery, or bronchoscopy). It is also not known whether infective triggers are associated with worsening prognosis. Then, we focus on AE associated with chronic fibrosing idiopathic interstitial pneumonias (AE-IIPs), including IPF, and evaluate differences in patient characteristics and examination results. Therefore, we purposed to evaluate prognosis for different characteristics and among idiopathic, infection-triggered, and non-infection-triggered AE-IIPs.

## Methods

### Patient selection and evaluations

We collected data for chronic fibrosing IIPs (CF-IIPs) patients who were hospitalized by respiratory failure between April 2009 and March 2019 at Juntendo University Hospital. We defined patients with CF-IIPs as having IPF, idiopathic non-specific interstitial pneumonia (NSIP), and idiopathic unclassifiable interstitial pneumonia (UCIP) based on the international IIPs classification by American thoracic society (ATS) and European respiratory society and a previous report [18, 19]. Initially, we excluded patients who presented with complications of congestive heart failure (CHF) or pneumothorax/mediastinum emphysema. From the remaining pool, we further excluded those with advanced lung cancer because we considered that the prognosis of patients with advanced lung cancer to be shorter than that of patients with CF-IIPs. Thereafter, we categorized the remainder of the patients into those with pneumonia superimposed on CF-IIPs and patients with AE-IIPs. Subsequently, we evaluated the difference in patient characteristics and prognosis between patients with pneumonia superimposed on CF-IIPs and those with AE-IIPs. Moreover, patients who developed AE-IIPs were included and classified into three clinical AE-IIPs types such as: idiopathic, infection-triggered, and non-infection-triggered AE-IIPs. We evaluated differences among the three groups in patient characteristics, baseline CF-IIPs pattern, baseline pulmonary function performed within 6 months from the development of AE-IIPs, pre-exiting CF-IIPs pattern, treatment prior to AE-IIPs, PaO_2_/FiO_2_ ratio, laboratory data (including white serum blood cell count, lactate dehydrogenase (LDH), C-related protein [CRP], Krebs von den Lungen-6 [KL-6], and surfactant protein-D [SP-D]), HRCT patterns at time of diagnosis of AE-IIPs, survival time, mortality, and seasonal variations. We evaluated HRCT findings at the time of diagnosis of AE-IIPs according to Akira’s previous publication, defining them as diffuse, multifocal, and peripheral patterns [20]. Three physicians (M.K., R.K., and S.S.) blindly reviewed the HRCT scans prior to the development of AE-IIPs and at the onset of AE-IIPs. Subsequently, we also evaluated risk factors for early death induced by AE-IIPs. Further, we defined early death as death within 30 days of onset.

### Defining AE-IIPs and triggers

AE-IIPs was diagnosed based on the ATS criteria (2016) [2] as follows: previous or concurrent diagnosis of any CF-IIPs, acute worsening or development of dyspnea typically of 1 month duration, and HRCT with new bilateral GGO and/or consolidation, superimposed on a background pattern associated with CF-IIPs and deterioration that is not fully explained by cardiac failure or fluid overload. We then divided patients who developed AE-IIPs into three groups based on the attending physician’s decision: idiopathic, infection-triggered, and non-infection-triggered. Moreover, we confirmed the findings associated with triggers as follows.

Post-operative AE-IIPs were defined as having been induced by any operation performed in an operating room that developed within a month after surgery. Drug-induced AE-IIPs were defined as having occurred after administration of the suspected drug and presence of previous reports of a drug-induced lung injury that developed within 3 months after drug initiation. Post-lung biopsy AE-IIPs were defined as those induced by bronchoscopy or percutaneous lung biopsy that developed within a month after the examinations. Infection-triggered AE-IIPs were defined as those induced by infection of the upper respiratory tract, contracted within 2 weeks prior to development of AE-IIPs, presenting with symptoms associated with upper respiratory tract inflammation, including high fever (> 37.5 degree, sore throat, worsening cough and worsening dyspnea, and/or virus positive, particularly influenza virus, or evidence of other infection. Bacterial pneumonia superimposed on CF-IIPs was defined as consolidation of the baseline interstitial shadow on HRCT images with evidence of bacterial infection, including positive serum procalcitonin, and/or positive smear or cultures of sputum and/or blood. We then distinguished between infection-triggered AE-IIPs and bacterial pneumonia superimposed on CF-IIPs based on attending physician’s decision. Patients with bacterial pneumonia superimposed on CF-IIPs were diagnosed by confirming elevated CRP and/or procalcitonin levels without elevated LDH, KL-6, and SP-D levels, and a consolidation area larger than the GGO area on HRCT. These criteria hold true except in cases of pneumothorax, pulmonary embolism or CHF, renal failure, and cases for which the main etiology was bacterial pneumonia.

We also evaluated seasonal variations among the three clinical AE-IIPs types. Seasons were defined as follows: between March and May as spring, between June and August as summer, between September and November as autumn, and between December and February as winter.

### Statistical analysis

We used the chi-square test, Fisher’s exact test, or Wilcoxon two-sample test to evaluate the frequency of any types of acute exacerbation and compare patient characteristics among these groups. Parametric and non-parametric data were compared with Student’s t-test and the Mann-Whitney U test, respectively. Differences in median survival time (MST) were analyzed with the log-rank test. Cox proportional hazard analysis was used to calculate hazard ratios (HRs), and univariate and multivariate logistic regression analyses were used to determine the risk factors for the AE-IIPs. A *p*-value of < 0.05 was considered statistically significant. All statistical analyses were performed with SPSS version 19.0 for Windows (Chicago, IL, USA).

## Results

### Patient characteristics

**Fig. 1:**
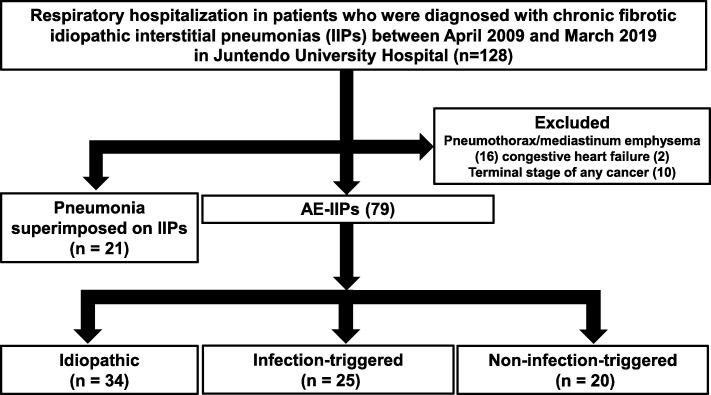
Flowchart of patient recruitment

We identified 128 CF-IIPs patients who were hospitalized by respiratory failure. From these patients, we excluded patients with pneumothorax or mediastinal emphysema (*n* = 16), CHF (*n* = 2), or terminal stage of any cancer (*n* = 10). Among them, 21 patients developed bacterial pneumonia superimposed on CF-IIPs; thus, we included 79 patients with CF-IIPs and with clinically diagnosed AE-IIPs (Fig. 1). Furthermore, patients were classified into three clinical types of AE-IIPs: idiopathic (*n* = 34), infection-triggered (*n* = 25), and non-infection-triggered (*n* = 20). Among the patients with infection-triggered AE-IIPs, 5 patients had a possible influenza infection, and all patients had symptoms related to upper respiratory tract infection, high fever or sore throat. Among patients who developed non-infection-triggered AE-IIPs, 12 had a history of new drug initiation, 5 had recently undergone operations, and 3 had received recent bronchoscopy or percutaneous lung biopsy. Patients with drug-induced AE-IIPs had received anti-cancer drugs (*n* = 6), including paclitaxel (*n* = 2), gemcitabine (*n* = 1), doxolbicine (*n* = 1), and axitinib (*n* = 1), antihypertensives (amlodipine) (*n* = 1), antibiotics (levofloxacin) (*n* = 1), anti-inflammatory drugs (*n* = 2), including loxoprofen (*n* = 1) and ibuprofen (*n* = 1), and herbal medications (*n* = 2), including Bofu-tsusho-san (*n* = 1) and Sho-saiko-to (*n* = 1).

Table 1 presents the patient characteristics. All patients were Japanese, with a median age of 74 years (range: 62–85 years). There were 66 men (83.52%). Sixty-one patients (77.22%) had a history of smoking, and 13 patients (16.46%) smoked until the development of AE-IIPs. Fifty-nine patients were diagnosed with IPF and 20 had UCIP prior to the development of AE-IIPs. There were no significant differences in patients characteristics among the three clinical AE-IIPs types. Prior to the development of AE-IIPs, 47 patients had received no treatment for CF-IIPs, while there were 9,6,9,4, and 4 patients treated with corticosteroids, corticosteroid plus immunosuppressants, pirfenidone, nintedanib, and corticosteroid plus nintedanib, respectively. All patients first received 1 g of methylprednisolone and 1 mg/kg of corticosteroid after steroid pulse therapy. There were no significant differences in prior treatment for baseline CF-IIPs between the groups (Table 1).

**Table 1:** Patient characteristics

Characteristics	Total *n* = 79	Idiopathic *n* = 34	Infection triggered *n* = 25	Non-infection triggered *n* = 20	*p*
Patients’ characteristics					
Age	74 (62 to 85)	75 (64 to 86)	75 (63 to 82)	72 (62 to 85)	*0.876*
Sex	66	29	19	18	*0.507*
Men (%)	83.54%	85.29%	76%	90%	
Smoking history	61	25	18	18	*0.271*
Yes (%)	77.22%	73.53%	72%	90%	
Current smoker	13	5	4	4	*0.592*
(%)	16.46%	14.71%	16%	20%	
Symptoms					
High fever	43	15	18	10	*0.094*
Yes (%)	54.43%	44.12%	72%	50%	
Sore throat	35	8	22	5	*0.0001*
Yes (%)	44.31%	23.52%	88%	25%	
Worsening cough	50	22	16	12	*0.808*
Yes (%)	63.29%	64.77%	64%	60%	
Worsening dyspnea	75	32	24	19	*0.926*
Yes (%)	94.94%	94.12%	96%	95%	
Pre-existing CF-IIPs					*0.072*
IPF	59	25	19	15	
UCIP	20	9	6	5	
Prior treatment					*0.177*
No treatment	47	17	14	16	
Corticosteroid	9	3	2	4	
Corticosteroid + IS	6	4	2	0	
Pirfenidone	9	4	5	0	
Nintedanib	4	3	1	0	
Corticosteroid + Nintedanib	4	3	1	0	
HRCT pattern at AE-IIPs onset					*0.471*
Diffuse	34	17	7	10	
Multifocal	14	7	5	2	
Peripheral	31	10	13	8	
Pulmonary function					
P/F ratio	168 (83.2 to 344.1) *n* = 79	173.7 (54.4 to 380.9) *n* = 34	213.3 (46.7 to 476.7) *n* = 25	134.2 (55.5 to 458.6) *n* = 20	*0.201*
FVC (L)	2.35 (0.94 to 3.01) *n* = 39	2.29 (1.53 to 3.07) *n* = 18	1.51 (0.78 to 2.76) *n* = 11	2.78 (1.94 to 4.45) *n* = 10	*0.002*
%FVC (%)	65.55 (39.85 to 84.02) *n* = 39	64.90 (47.20 to 82.66) *n* = 18	51.33 (33.34 to 81.62) *n* = 11	73.70 (57.00 to 87.90) *n* = 10	*0.008*
VC (L)	2.49 (1.33 to 3.05) *n* = 39	2.42 (1.43 to 3.15) *n* = 18	1.64 (0.83 to 3.16) *n* = 11	2.85 (2.08 to 4.32) *n* = 10	*0.002*
%VC (%)	70.70 (44.60 to 90.22) *n* = 39	67.35 (47.00 to 93.18) *n* = 18	52.00 (35.16 to 81.33) *n* = 11	81.11 (59.91 to 119.41) *n* = 10	*0.004*
DLco (mL/min/mmHg)	(3.85 to 11.35) *n* = 30	6.44 (3.73 to 12.53) *n* = 16	7.74 (2.92 to 11.51) *n* = 7	8.04 (4.08 to 10.09) *n* = 7	*0.959*
%DLco (%)	29.90 (16.55 to 40.02) *n* = 30	25.35 (16.05 to 52.77) *n* = 16	33.70 (12.40 to 45.50) *n* = 7	32.41 (18.11 to 44.42) *n* = 7	*0.951*
Serum markers					
KL-6 (IU/L)	1117 (572 to 2701) *n* = 79	1312 (692 to 2663) *n* = 34	1019 (222 to 3509) *n* = 25	1372 (659 to 3528) *n* = 20	*0.166*
SP-D (mg/dL)	364 (45 to 2711) *n* = 63	370 (77 to 2430) *n* = 29	320 (20 to 1170) *n* = 20	370 (133 to 3180) *n* = 14	*0.328*
LDH (IU/L)	365 (193 to 539) *n* = 79	346 (193 to 546) *n* = 34	327 (201 to 765) *n* = 25	436 (175 to 652) *n* = 20	*0.060*
WBC (/μL)	10,700 (6300 to 15,400) *n* = 79	10,400 (6200 to 17,880) *n* = 34	10,600 (5700 to 22,900) *n* = 25	12,600 (2500 to 22,900) *n* = 20	*0.918*
CRP (mg/dL)	8.5 (0.7 to 25) *n* = 79	7.3 (0.7 to 22) *n* = 34	11.1 (0.87 to 36) *n* = 25	7.5 (1.3 to 25.3) *n* = 20	*0.063*
Procalcitonin (ng/mL)	0.12 (0.01 to 0.74) *n* = 70	0.15 (0.01 to 0.58) *n* = 32	0.33 (0.07 to 0.74) *n* = 21	0.09 (0.01 to 0.31) *n* = 17	*0.037*
D-dimer (mg/dL)	3.6 (1.38 to 74.2) *n* = 74	3 (1.8 to 83) *n* = 31	2.6 (1.3 to 21.1) *n* = 24	6.1 (1.2 to 41.0) *n* = 19	*0.222*

*Abbreviations*: *CF-IIPs* Chronic fibrosing idiopathic interstitial pneumonias, *IPF* Idiopathic pulmonary fibrosis, *UCIP* Unclassifiable interstitial pneumonia, *IS* Immunosuppressant, AE-IIPs Acute exacerbation of idiopathic interstitial pneumonias, *P/F ratio* PaO_2_/FiO_2_ ratio, *FVC* Forced vital capacity, *VC* Vital capacity, *DLco* Diffusing capacity for carbon monoxide, *KL-6* Krebs von den Lungen-6, *SP-D* Surfactant protein-D, *LDH* Lactate dehydrogenase, *WBC* White blood cells, *CRP* C-related protein

In addition, comparison of patient backgrounds between patients with AE-IIPs and those with pneumonia superimposed on CF-IIPs showed no significant differences in parameters, including age, sex, smoking history, baseline CF-IIPs pattern, and prior treatment for CF-IIPs (Table 2).

**Table 2:** The difference of patient characteristics, pulmonary function, and serum markers between AE-IIPs and pneumonia

	AE-IIPs *n* = 79	Pneumonia superimposed on IIPs *n* = 21	*p*
Patient characteristics			
Age	74 (62 to 85)	75 (66 to 83)	*0.869*
Sex	66	18	*0.807*
Men (%)	83.54%	85.71%	
Smoking history	61	16	*0.921*
Yes (%)	77.22%	76.19%	
Current smoker	13	3	*0.357*
(%)	16.46%	14.29%	
Symptoms			
High fever	43	17	*0.027*
Yes (%)	54.43%	80.91%	
Sore throat	35	19	*0.008*
Yes (%)	44.31%	90.48%	
Worsening cough	50	15	*0.661*
Yes (%)	63.29%	71.42%	
Worsening dyspnea	75	18	*0.321*
Yes (%)	94.94%	85.72%	
Pre-existing CF-IIPs			*0.542*
IPF	59	17	
UCIP	20	4	
Prior treatment			*0.248*
No treatment	47	8	
Corticosteroid	9	2	
Corticosteroid + IS	6	2	
Pirfenidone	9	2	
Nintedanib	4	4	
Corticosteroid + Nintedanib	4	3	
Pulmonary function			
P/F ratio	168 (83.2 to 344.1) *n* = 79	246 (173.7 to 340.5) *n* = 18	*0.026*
FVC (L)	2.35 (0.94 to 3.01) *n* = 39	2.19 (1.43 to 3.22) *n* = 17	*0.807*
%FVC (%)	65.55 (39.85 to 84.02) *n* = 39	70.11 (41.98 to 102.22) *n* = 17	*0.356*
VC (L)	2.49 (1.33 to 3.05) *n* = 39	2.09 (1.24 to 3.04) *n* = 17	*0.266*
%VC (%)	70.70 (44.60 to 90.22) *n* = 39	65.21 (40.02 to 101.61) *n* = 17	*0.734*
DLco (mL/min/mmHg)	7.29 (3.85 to 11.35) *n* = 30	5.04 (2.37 to 8.73) *n* = 17	*0.006*
%DLco (%)	29.90 (16.55 to 40.02) *n* = 30	21.00 (10.71 to 40.02) *n* = 17	*0.014*
Serum markers			
KL-6 (IU/L)	1117 (572 to 2701) *n* = 79	517 (296 to 2348) *n* = 21	*0.003*
SP-D (mg/dL)	364 (45 to 2711) *n* = 63	107 (44 to 303) *n* = 17	*0.006*
LDH (IU/L)	365 (193 to 539) *n* = 79	231 (157 to 359) *n* = 21	*0.001*
WBC (/μL)	10,700 (6300 to 15,400) *n* = 79	9900 (5480 to 17,880) *n* = 21	*0.496*
CRP (mg/dL)	8.5 (0.7 to 25) *n* = 79	16.4 (2.2 to 35.8) *n* = 21	*0.011*
Procalcitonin (ng/mL)	0.12 (0.01 to 0.74) *n* = 70	1.86 (0.46 to 11.21) *n* = 19	*0.001*
D-dimer (mg/dL)	3.6 (1.38 to 74.2) *n* = 74	2.3 (1.2 to 4.1) *n* = 19	*0.006*

*Abbreviations*: *IIPs* Idiopathic interstitial pneumonias, *AE-IIPs* Acute exacerbation of idiopathic interstitial pneumonias, *IPF* Idiopathic pulmonary fibrosis, *UCIP* Unclassifiable interstitial pneumonia, *P/F ratio* PaO_2_/FiO_2_ ratio, *FVC* Forced vital capacity, *VC* Vital capacity, *DLco* Diffusing capacity for carbon monoxide, *KL*-6 Krebs von den Lungen-6, *SP-D* Surfactant protein-D, *LDH* Lactate dehydrogenase, *WBC* White blood cells, *CRP* C-related protein

### AE-IIPs characteristics

HRCT indicated a diffuse pattern in 34 patients, a multifocal pattern in 14 patients, and a peripheral pattern in 31 patients at the time of diagnosis of AE-IIPs. There were no significant differences in HRCT patterns associated with AE-IIPs among the three groups. For AE-IIPs-associated clinical symptoms, including high fever (> 37.5 degree), worsening cough, and worsening dyspnea, the number of patients with high fever tended to be higher in infection-triggered AE-IIPs than in other clinical AE-IIPs types (*p* = 0.094). For other symptoms related to AE-IIPs or CF-IIPs, including worsening cough and dyspnea, there were no significant differences in these symptoms among these groups. In contrast, there were no significant differences in other symptoms, including worsening cough and dyspnea.

Among the serum markers, although serum CRP in infection-triggered patients was significantly higher than other clinical AE-IIPs types, the serum procalcitonin levels was significantly higher in infection-triggered AE-IIPs than in patients with all total AE-IIPs. Then, the serum procalcitonin was significantly higher in infection-triggered AE-IIPs than in idiopathic AE-IIPs (*p* = 0.037). Moreover, serum LDH levels tended to be higher in non-infection triggered patients than those in other types of AE-IIPs. In contrast, there were no significant differences in other serum markers, including KL-6 and SP-D, as well as the PaO_2_/FiO_2_ ratio among the three groups. Among pulmonary function measures, although forced vital capacity (FVC) and vital capacity (VC) were significantly higher in non-infection-triggered AE-IIPs, there was no significant difference in diffusion capacity (DLco) among the three groups (Table 1).

In addition, symptoms, serum marker levels, and pulmonary function were compared between patients with pneumonia superimposed on CF-IIPs and those with AE-IIPs. The number of patients with high fever and sore throat in the bacterial pneumonia group were significantly higher than those in the AE-IIPs group (*p* = 0.027 and *p* = 0.008, respectively).Although there was no significant difference in the serum WBC counts, serum KL-6, SP-D, and D-dimer levels in patients with AE-IIPs were significantly higher than those with pneumonia superimposed on CF-IIPs. In contrast, serum CRP and procalcitonin level was significantly lower in patients with AE-IIPs than in those with pneumonia superimposed on CF-IIPs. In addition, the serum procalcitonin was significantly higher in bacterial pneumonia superimposed on CF-IIPs than in infection-triggered AE-IIPs (*p* = 0.001).

Moreover, the baseline pulmonary function parameter, DLco, was significantly lower in patients with pneumonia superimposed on CF-IIPs than in those with AE-IIPs, whereas there were no significant differences in FVC and VC between the two groups. An echocardiography was performed to evaluate left and/or right heart failure and pulmonary embolism in all patients diagnosed with AE-IIPs, including the patients with elevated D-dimer levels. No patients were found to have left/right heart failure and embolism using an echocardiography (Table 2).

All patients with AE-IIPs were treated with intravenous high-dose corticosteroids and an antibiotic. Approximately 40% of these patients were also treated with immunosuppressants. We performed invasive ventilation with only a few patients but administered high-flow therapy to over 60% of the patients. However, there were no significant differences in treatment and use of ventilation among the different types of AE-IIPs (Table 3). When comparing the difference in treatment between patients who were diagnosed with AE-IIPs and those who were diagnosed with bacterial pneumonia superimposed on CF-IIPs, although all patients received antibiotics for both AE-IIPs and bacterial pneumonia superimposed on CF-IIPs, no patients received additional corticosteroids and/or immunosuppressants in the bacterial pneumonia group.

**Table 3:** The difference of treatment for AE-IIPs

Treatment	Total *n* = 79	Idiopathic *n* = 34	Infection-triggered *n* = 25	Non-infection-triggered *n* = 20	*p*
Admission to intensive care unit	55	24	17	14	*0.796*
	69.62%	70.59%	68%	70%	
Only intensive care unit admission	13	7	2	4	*0.325*
	16.46%	20.59%	8%	20%	
Intravenous high-dose steroids	79	34	25	20	*1.000*
	100%	100%	100%	100%	
Immunosuppressant	34	17	9	8	*0.413*
	43.03%	50%	36%	40%	
Antibiotics	79	34	25	20	*1.000*
	100%	100%	100%	100%	
Invasive ventilation	7	3	1	3	*0.386*
	8.86%	8.82%	4%	15%	
NPPV	14	6	4	4	*0.894*
	17.72%	17.65%	16%	20%	
High-flow therapy	51	25	12	14	*0.103*
	64.56%	73.53%	48%	70%	

*Abbreviations*: *NPPV* Non-invasive positive pressure ventilation

**Fig. 2:**
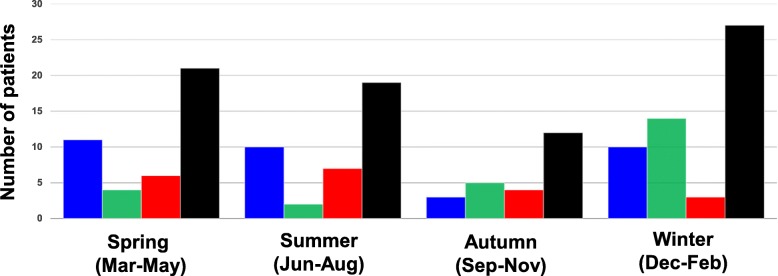
Differences in seasonal incidence of AE-IIPs among the three groups. Blue, green, red, and black bars indicate the number of patients with idiopathic, infection-triggered, non-infection-triggered, and total AE-IIPs, respectively

We also analyzed seasonal variations in the development of AE-IIPs. Among patients with infection-triggered AE-IIPs, 4 developed AE-IIPs in spring, 1 in summer, 5 in autumn, and 14 in winter, representing a significantly higher incidence in winter than in any other season (*p* = 0.035). However, there were no significant differences in the incidence of idiopathic and non-infection-triggered AE-IIPs between any seasons (Fig. 2).

### Risk factors associated with AE-IIPs-induced early death

Tables 4 and 5 present the results of univariate and multivariate analyses of risk factors for early death associated with AE-IIPs. We defined early death as death within 30 days of development of AE-IIPs. In the univariate analysis, PaO_2_/FiO_2_ ratio, HRCT patterns at AE-IIPs onset, treatment prior to AE-IIPs, and clinical AE-IIPs types were significantly associated with early death (PaO_2_/FiO_2_ ratio: OR = 11.511, *p* = 0.0007; HRCT patterns at AE-IIPs onset: OR = 16.011, *p* = 0.0003; and clinical AE-IIPs types: OR = 13.138, *p* = 0.0009). Multivariate analysis performed using three variables (PaO_2_/FiO_2_ ratio, HRCT patterns at AE-IIPs onset, and AE-IIPs types) indicated that HRCT patterns at AE-IIPs onset and clinical AE-IIPs types were significant independent risk factors for early death [clinical AE-IIPs types: OR = 2.375, 95% confidence interval (95%CI) = 1.173–4.807, *p* = 0.016; and HRCT patterns at AE-IIPs onset: OR = 2.032, 95%CI = 1.128–3.663, *p* = 0.018].

**Table 4:** The difference of patient characteristics, pulmonary function, and serum markers between survivors and non-survivors

Variables	Overall	survivor	Non-survivor	OR	*p*
	*n* = 79	*n* = 42	*n* = 37		
Patient background					
Age	74 (62 to 86)	75 (64 to 86)	74 (62 to 85)	1.113	*0.374*
Sex	66	32	34	3.333	*0.126*
Man (%)	83.54%	76.19%	91.89%		
Smoking history	61	31	30	1.428	*0.597*
Yes (%)	77.22%	73.81%	81.08%		
Current smoker	13	7	6	1.128	*0.973*
Yes (%)	16.46%	16.67%	14.29%		
Time to diagnosis of IIPs	33.25 ± 38.39	31.20 ± 35.24	37.76 ± 42.36	2.176	*0.124*
Symptoms					
High fever	43	23	20	1.012	*0.949*
Yes (%)	54.43%	54.76%	54.05%		
Sore throat	35	22	13	3.841	*0.132*
Yes (%)	44.13%	52.38%	35.14%		
Worsening cough	50	27	23	1.011	*0.969*
Yes (%)	63.29%	64.28%	62.16%		
Worsening dyspnea	75	39	36	1.618	*0.701*
Yes (%)	94.94%	92.85%	97.29%		
Pre-existing CF-IIPs				0.409	*0.815*
IPF	59	33	26		
UCIP	20	9	11		
Prior treatment				2.793	*0.593*
No treatment	47	28	19		
Corticosteroid	9	4	5		
Corticosteroid + IS	6	2	4		
Pirfenidone	9	5	4		
Nintedanib	4	2	2		
Corticosteroid + Nintedanib	4	1	3		
Clinical AE-IIPs types				13.138	*0.0009*
Idiopathic	34	12	22		
Infection-triggered	25	20	5		
Non-infection-triggered	20	10	10		
HRCT patterns at AE-IIPs onset				16.011	*0.0003*
Diffuse	34	9	25		
Multifocal	14	12	2		
Peripheral	31	21	10		
Pulmonary function					
P/F ratio	168 (83.2 to 344.1) *n* = 79	246 (103 to 362) *n* = 42	132 (67 to 339) *n* = 37	11.510	*0.0007*
VC (%)	2.49 (1.33 to 3.05) *n* = 39	2.43 (0.95 to 3.45) *n* = 23	2.58 (1.87 to 3.45) *n* = 16	2.329	*0.127*
VC (L)	70.70 (44.60 to 90.22) *n* = 39	69.55 (38.50 to 93.55) *n* = 23	71.21 (48.63 to 99.74) *n* = 16	0.655	*0.418*
FVC (%)	2.35 (0.94 to 3.01) *n* = 39	2.35 (0.89 to 3.45) *n* = 23	2.38 (1.84 to 3.47) *n* = 16	2.212	*0.136*
FVC (L)	65.55 (39.85 to 84.02) *n* = 39	65.33 (35.58 to 86.82) *n* = 23	67.51 (50.20 to 86.00) *n* = 16	1.084	*0.297*
DLco (mL/min/mmHg)	7.29 (3.85 to 11.35) *n* = 30	7.63 (4.33 to 11.76) *n* = 16	6.79 (3.68 to 11.45) *n* = 14	0.080	*0.776*
DLco (%)	29.90 (16.55 to 40.02) *n* = 30	31.5 (17.68 to 51.08) *n* = 16	26.35 (15.75 to 44.70) *n* = 14	0.356	*0.551*
Serum markers					
KL-6 (IU/L)	1117 (572 to 2701) *n* = 79	1086 (569 to 2882) *n* = 42	1166 (583 to 2585) *n* = 37	28.571	*0.938*
SP-D (mg/dL)	364 (45 to 2711) *n* = 63	362 (148 to 987) *n* = 36	370 (76 to 1382) *n* = 27	1.612	*0.424*
LDH (IU/L)	365 (193 to 539) *n* = 79	350 (249 to 539) *n* = 42	368 (228 to 540) *n* = 37	5.586	*0.671*
WBC (/μL)	10,700 (6300 to 15,400) *n* = 79	10,690 (5960 to 15,900) *n* = 42	11,300 (8600 to 14,600) *n* = 37	4.037	*0.620*
CRP (mg/dL)	8.5 (0.7 to 25) *n* = 79	7.5 (1.98 to 24.65) *n* = 42	8.8 (1.86 to 21.76) *n* = 37	37.037	*0.987*
Procalcitonin (ng/mL)	0.12 (0.01 to 0.74) *n* = 70	0.12 (0.01 to 0.74) *n* = 38	0.10 (0.01 to 0.68) *n* = 32	1.021	*0.878*
D-dimer (mg/dL)	3.6 (1.38 to 74.2) *n* = 74	3.1 (1.27 to 17.18) *n* = 41	4.9 (1.88 to 33.32) *n* = 33	2.002	*0.157*

*Abbreviations*: *OR* Odds ratio, *CF-IIPs* Chronic fibrosing idiopathic interstitial pneumonias, *IPF* Idiopathic pulmonary fibrosis, *UCIP* Unclassifiable interstitial pneumonia, *P/F ratio* PaO_2_/FiO_2_ ratio, *FVC* Forced vital capacity, *VC* Vital capacity, *DLco* Diffusing capacity for carbon monoxide, *KL-6* Krebs von den Lungen-6, *SP-D* Surfactant protein-D, *LDH* Lactate dehydrogenase, *WBC* White blood cells, *CRP* C-related protein

**Table 5:** Multivariate analysis of risk factors associated with AE-IIPs-induced death within 30 days

Variable	OR	95% CI	*p*
P/F ratio	0.989	0.983–0.996	*0.0001*
Clinical AE-IIPs types	2.375	1.173–4.807	*0.016*
HRCT patterns at AE-IIPs onset	2.032	1.128–3.663	*0.018*

*Abbreviations*: *OR* Odds ratio, *95%CI* 95% confidence interval, *P/F ratio* PaO_2_/FiO_2_ ratio, *AE-IIPs* Acute exacerbation associated with idiopathic interstitial pneumonias, *HRCT* High resolution computed tomography

### Prognostic differences among AE-IIPs groups

**Fig. 3:**
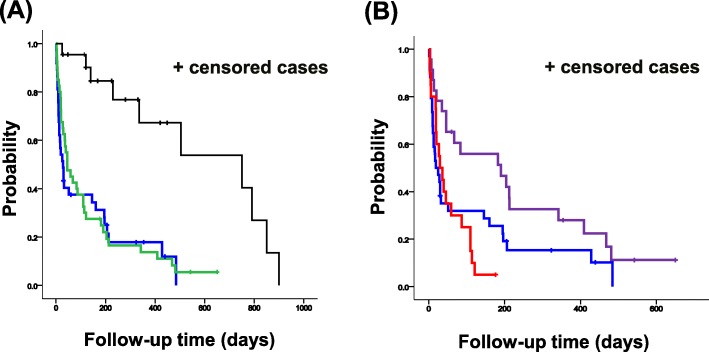
Survival curves. **a** Survival time from development of AE-IIPs or pneumonia among idiopathic, all triggered (infection-triggered and non-infection-triggered) AE-IIPs, and pneumonia superimposed on CF-IIPs. Blue, green, and black lines indicate the number of patients and survival for idiopathic, all triggered AE-IIPs, and pneumonia superimposed on CF-IIPs, respectively. *p*-value was calculated using the log-rank test. **b** Difference in survival time from AE-IIPs onset. Blue, red, and purple lines indicate the number of patients and survival for idiopathic, non-infection-triggered, infection-triggered AE-IIPs, respectively. *p*-value was calculated using the log-rank test

We evaluated differences in survival among the different types of AE-IIPs and pneumonia superimposed on CF-IIPs. Although there was no significant difference in MST between idiopathic and all triggered AE-IIPs (idiopathic: 19 days, 95%CI = 2.119–35.881; all triggered: 46 days, 95%CI = 15.048–76.952), MST was significantly longer in patients with pneumonia superimposed on CF-IIPs (750 days, 95%CI = 248.328–1251.672) than that in patients with all triggered or idiopathic AE-IIPs (HR = 25.137, *p* = 0.0001, Fig. 3a). Moreover, MST was significantly longer in patients with infection-triggered exacerbations (190 days, 95%CI = 10.157–369.853) than that in patients with idiopathic or non-infection-triggered AE-IIPs (29 days, 95%CI = 12.057–43.493, *p* = 0.012, Fig. 3b).

Finally, we analyzed the mortality rate 15, 30, 60, and 90 days after AE-IIPs onset. On all observation days, the mortality rate was significantly lower in infection-triggered AE-IIPs than in idiopathic and non-infection-triggered AE-IIPs. In addition, we also analyzed differences in cause of death among the three groups. Although AE-IIPs itself was the most common cause of death in idiopathic and non-infection-triggered patients, chronic respiratory failure was the most common cause of death in infection-triggered AE-IIPs (Table 6). In addition, there were 5 patients (2 with idiopathic, 2 with non-infection-triggered, and one with infection-triggered) who developed recurrent AE-IIPs. There was no significant association between recurrence and clinical AE-IIPs types among three groups.

**Table 6:** Mortality and cause of death

	Total *n* = 79	Idiopathic *n* = 34	Infection-triggered *n* = 25	Non-infection-triggered *n* = 20	*p*
Mortality					
14 days	22 27.84%	14 41.18%	4 16%	4 20%	*0.049*
28 days	37 46.83%	22 64.71%	5 20%	10 50%	*0.029*
56 days	41 51.89%	21 61.76%	8 32%	12 60%	*0.025*
90 days	50 63.29%	24 70.59%	11 44%	15 75%	*0.022*
The number of already death patients	70 88.61%	29 85.29%	22 88%	19 95%	
Cause of death					
AE-IIPs	40 57.97%	25 86.21%	5 23.81%	10 52.63%	*0.0003*
Bacterial pneumonia	6 8.67%	1 3.45%	4 19.05%	1 5.26%	
Chronic respiratory failure	18 26.09%	3 10.34%	9 42.86%	6 31.57%	
Others	6 8.67%	0 0%	4 19.05%	2 10.52%	

*Abbreviations*: *AE-IIPs* Acute exacerbation of idiopathic interstitial pneumonias

## Discussion

To our knowledge, the current study is the first to classify AE-IIPs into three groups (idiopathic, infection-triggered, and non-infection-triggered AE-IIPs) and to evaluate differences in prognosis among the three groups. Our main findings are as follows: (i) survival time was significantly longer in patients with infection-triggered AE-IIPs than in patients with idiopathic or non-infection-triggered AE-IIPs, and moreover, the survival time was significantly longer in patients with pneumonia superimposed on CF-IIPs than in patients with AE-IIPs; (ii) mortality rate was significantly lower in infection-triggered AE-IIPs than in either other type; (iii) the incidence of infection-triggered AE-IIPs in winter was significantly higher than in any other season; (iv) variation in AE-IIPs was not significantly associated with HRCT patterns at AE-IIPs onset and previous treatment; and (v) clinical AE-IIPs types and HRCT patterns at AE-IIPs onset were significant independent risk factors for early death induced by AE-IIPs.

Recently, several Japanese publications have reported differences in prognosis between idiopathic and triggered AE-IIPs [21, 22]. However, these differences were not significant. Although there are several different triggers, including infection, drugs, surgery, anesthesia, and bronchoscopy, no previous studies have evaluated whether there are differences in prognosis and patient characteristics among the different triggers. Thus, we focused on the kind of triggers, specifically the presence or absence of infections, and evaluated differences in prognosis among idiopathic, infection-triggered, and non-infection-triggered AE-IIPs.

In terms of prognosis, MST for infection-triggered AE-IIPs was the longest, followed by idiopathic, and then non-infection-triggered AE-IIPs. Moreover, there was a significant difference in the most common cause of death between infection-triggered and non-infection-triggered/idiopathic AE-IIPs. All patients received antibiotics. Therefore, control of infection may be associated with attenuation of infection-related inflammation and injury, thereby improving the survival time in patients with infection-triggered AE-IIPs. In contrast, we suggest that for any triggers other than infection it is extremely difficult to inhibit or attenuate the lung injury induced by drugs, biopsy, surgery, or anesthesia because these triggers cause chemical or physical injury. Survival in patients with idiopathic AE-IIPs was intermediate between patients with infection-triggered and non-infection-triggered AE-IIPs.

Moreover, it may be also difficult to accurately classify idiopathic and infection-triggered AE-IIPs. In this study, we classified patients with symptoms related to upper respiratory tract inflammation, as having infection-triggered AE-IIPs. Corticosteroid treatment is known to interfere with the development of a fever. When a patient receives corticosteroids, they may not develop fever and other symptom associated with influenza virus infection. This patient would be classified as having the idiopathic type. Thus, treatment of baseline CF-IIPs may increase the selection bias, and idiopathic AE-IIPs may include unknown triggers that are both infection and non-infection related. Although the frequency of high fever was significantly higher in bacterial pneumonia superimposed on CF-IIPs patients than that in AE-IIPs, there were several patients who received corticosteroids in all categories and almost all of these patients did not have high fever in all clinical AE-IIPs types. Therefore, the data may be indeterminate for evaluating whether a high fever is associated with AE-IIPs in patients who received corticosteroids.

The evaluation of procalcitonin levels may be useful for the distinguishing among clinical AE-IIPs types, and between AE-IIPs and bacterial pneumonia. In this study, the levels of procalcitonin was significantly higher in patients with infection-triggered than in patients with other clinical AE-IIPs. Then, the levels of procalcitonin was significantly higher in patients with bacterial pneumonia superimposed on CF-IIPs than in patients with AE-IIPs, especially in infection-triggered AE-IIPs. Therefore, evaluation of procalcitonin levels may be reasonable to distinguish bacterial pneumonia and AE-IIPs and to classify among clinical AE-IIPs types. However, the patients could easily succumb to other bacterial after viral infection compared with healthy individuals; thus, only evaluating procalcitonin and/or CRP levels may not be useful for distinguishing among the clinical AE-IIPs types, and between AE-IIPs and bacterial pneumonia superimposed on CF-IIPs.

Further, whereas there was significant difference in survival time among three groups, the three survival curves almost overlapped immediately after the development of AE-IIPs. Therefore, we were unable to evaluate the prognosis and severity during from the early phase to the development of AE-IIPs, for distinguishing the clinical AE-IIPs types. In contrast, no patients with bacterial pneumonia superimposed on CF-IIPs were admitted to only the intensive care unit (ICU). In addition, the survival curve evidently differed between AE-IIPs and bacterial pneumonia superimposed on CF-IIPs from the early phase of AE-IIPs. Thus, we believe that the difference in ICU admission, which reflects on the severity, may be associated with the difference in clinical course and pathogenesis between AE-IIPs and bacterial pneumonia superimposed on CF-IIPs.

The major causes of death in IPF have been shown to be AE-IIPs, development of lung cancer, and chronic respiratory failure, based on previous reports [23, 24]. Respiratory failure is defined as a decrease in PaO_2_ and a > 45 Torr in Japan. Following the development of chronic respiratory failure in CF-IIPs patients, the patient’s quality of life decreases, and the risk of comorbidities associated with CF-IIPs increases. These patients die due to respiratory failure. In this study, there were no patients who died of lung cancer after the development of AE-IIPs. The difference in the cause of death between this study and the recent study [24] in total IPF was considered to be the difference in prognosis between the patients with and without AE-IIPs. It was reported that the survival time in patients with AE-IIPs was significantly shorter than that in patients without AE-IIPs. The difference in prognosis may associate with differences in the cause of death [20, 24].

The incidence of AE-IIPs in winter was only significantly higher in infection-triggered AE-IIPs compared with other types of AE-IIPs. In the INPULSIS trial, a phase 3 clinical trial of nintedanib for IPF, the incidence of AE-IIPs was also higher in winter than in other seasons [25]. In this study, AE-IIPs was not divided into idiopathic and triggered types. Consistent with this, another publication has reported that the incidence of AE-IIPs in winter is significantly higher than that in the other seasons [26]. However, although Teramachi et al. reported a higher AE-IIPs incidence in idiopathic patients in winter than in other seasons, there was no significant seasonal difference in patients with triggered AE-IIPs [21]. In general, AE-IIPs more commonly develop in winter than in other seasons because viral respiratory infections and other winter-specific factors may be associated with the development of AE-IIPs. This conclusion is supported by the results of the INPULSIS trial, as well as our current results.

This study is subject to several limitations. First, this was a small retrospective study. Although there was a moderate number of patients with CF-IIPs at our hospital, the incidence of AE-IIPs was approximately 10–20% per year [4, 24]. Therefore, it is difficult to recruit a large number of patients who have developed AE-IIPs. Second, almost patients were diagnosed and classified into each CF-IIPs based on HRCT findings, serological findings, and medical examination by an interview, but not on histological findings. Therefore, the patients’ backgrounds were heterogeneous, and survival may have resulted from selection bias. Then, although 3 patients showed a NSIP pattern (GGO and/or reticular shadow on the bronchovascular bundles) on the HRCT, these patients were diagnosed with UCIP due to presence of both UIP and NSIP patterns or other mixed pathological features. We considered it to be a coincidence that there were no patients diagnosed with NSIP by small group bias. Moreover, there were no significant differences in baseline IIPs types among the clinical AE-IIPs types and between AE-IIPs and bacterial pneumonia superimposed on CF-IIPs Thus, it may not be important to classify IIPs in detail to distinguish among the clinical AE-IIPs types and baseline IIPs types in order to predict the prognosis of AE-IIPs in our study. Third, it was strictly difficult to distinguish between infection-triggered AE-IIPs and development of bacterial pneumonia in patients with CF-IIPs. Although MST was significantly longer in bacterial pneumonia than in infection-triggered AE-IIPs, patients who only developed pneumonia may be accidentally confused with infection-triggered AE-IIPs. Fourth, in our study, we collected the data on pulmonary function within 6 months of the development of AE-IIPs. Since the pulmonary function results were over 6 months old, this was not appropriate for evaluating the association between the pulmonary function and AE-IIPs development. Therefore, we evaluated pulmonary function in only 39 patients. Evaluation of pulmonary function only using data from half of the total patients may result in a lower accuracy.

## Conclusion

Our findings suggest that patients with infection-triggered AE-IIPs have a better prognosis than do patients with other types of AE-IIPs.

## Data Availability

Not applicable.

## Abbreviations

95%CI: 95% confidence interval
AE: Acute exacerbation
AE-IIPs: Acute exacerbation of interstitial pneumonia
AE-IPF: Acute exacerbation of idiopathic pulmonary fibrosis
ATS: American Thoracic Society
CF-IIPs: Chronic fibrosing idiopathic interstitial pneumonias
CHF: Congestive heart failure
CRP: C-related protein
DLco: Diffusing capacity for carbon monoxide
FVC: Forced vital capacity
GGO: Ground-glass opacities
HR: Hazard ratio
HRCT: High resolution computed tomography
ICU: Intensive care unit
IIPs: Idiopathic interstitial pneumonias
IPF: Idiopathic pulmonary fibrosis
KL-6: Krebs von den Lungen-6
LDH: Lactate dehydrogenase
MST: Median survival time
NSIP: Non-specific interstitial pneumonia
OR: Odds ratio
P/F ratio: PaO2/FiO2 ratio
SP-D: Surfactant protein-D
UCIP: Unclassifiable interstitial pneumonia
VC: Vital capacity
